# Does a greater training load increase the risk of injury and illness in ultramarathon runners? : A prospective, descriptive, longitudinal design

**DOI:** 10.17159/2078-516X/2020/v32i1a8559

**Published:** 2020-01-01

**Authors:** N Craddock, K Buchholtz, T L Burgess

**Affiliations:** 1 Division of Physiotherapy, Department of Health and Rehabilitation Sciences, University of Cape Town, Old Main Building, Groote Schuur Hospital, Anzio Road, Observatory, Cape Town, South Africa; 2 Division of Exercise Science and Sports Medicine, Faculty of Health Sciences, University of Cape Town; 3 Department of Physiotherapy, LUNEX University, Luxembourg; 4 Centre for Medical Ethics and Law, Faculty of Medicine and Health Sciences, Stellenbosch University, Cape Town, South Africa

**Keywords:** running-related injuries and illness, acute:chronic workload ratio, exponentially-weighted moving average

## Abstract

**Background:**

Ultramarathon running has become extremely popular over the years. Despite the numerous health benefits of running, there are also many negative effects of running, such as increased risk of musculoskeletal injury and illness. Monitoring of an athlete’s training load has become extremely important in terms of injury prevention. Currently, the relationship between training loads and injury and illness incidence is uncertain.

**Objectives:**

To determine if there are any associations between injury and illness incidences and training loads among ultramarathon runners in the 12 week period preceding an ultramarathon event and the four week period after the event.

**Methods:**

This prospective, descriptive, longitudinal study design was conducted in 119 runners who were training for the 2019 Two Oceans ultramarathon event. Data were collected once a week via an online logbook over 16 weeks. Training parameters measured included weekly average running distance, average duration, average frequency and average sessional RPE. Injury data included injury counts, the structure injured, the main anatomical location and the time-loss as a result of injury. Illness data included illness counts, the main illness-related symptoms and the time-loss as a result of illness.

**Results:**

The overall injury incidence was five per 1000 training hours and the overall illness incidence was 16 per 1000 training days. There was a significant relationship between external training load and injury and illness incidence for those who ran less than 30 km per week. There was also a significant relationship between the ACWR (Acute Chronic Workload Ratio) and injury incidence when the ACWR was >1.5 and for illness incidence when the ACWR was <0.5.

**Conclusion:**

The use of both absolute and relative workloads in the monitoring of an athlete’s training load with the aim of minimising injury and illness risk and maximising performance in ultramarathon runners is recommended.

Endurance running has become increasingly popular over the past three decades due to its numerous health benefits and relative accessibility ^[1]^. In particular, there has been a significant rise in participation in ultramarathon running events. Despite the positive benefits of running, endurance running has been associated with an increased risk of developing musculoskeletal injury ^[2]^. This is evidenced by an overall running-related injury incidence rate ranging from between 18% and 92% in ultramarathon runners ^[3]^. In addition to this association with injury, ultramarathon running seems to also influence illness risk. While consistent, moderate-intensity exercise has beneficial effects on general health and immune system function, prolonged high-intensity exercise has been shown to impair immune system function and thus increase the risk of acquiring an upper respiratory tract infection ^[4]^.

The training load of endurance runners could potentially have a profound effect on the development of both running-related injury and illness ^[5]^. Training is performed in order to bring about positive physiological adaptations in preparation for an athlete’s sporting endeavour, with the aim of maximising performance. However, it is hypothesised that too great a training load could predispose an athlete to injury and illness. In contrast, too small a training load could possibly lead to inadequate conditioning for the requirements of the sport, and thus result in injury and reduced performance ^[5]^. Therefore, finding the optimal training load to maximise performance, whilst minimising injury and illness risk, should be the goal of both coaches and athletes.

Training load as defined by Gabbett is the combination of both the absolute load (internal and external training load) and the relative load (week to month ratio) ^[5]^. The internal load of a training session can be calculated as duration multiplied by rating of perceived exertion (RPE) whilst the external training load refers to distance, duration, intensity and frequency ^[6]^. The acute:chronic workload ratio (ACWR) describes the acute load in relation to the chronic load. ‘Acute’ is one week in duration and ‘chronic’ can be three to six weeks in duration ^[5]^. Acute loading is comparable to a state of fatigue and chronic loading is comparable to a state of fitness. Therefore, the ratio tells us how ‘prepared’ the athlete is and the relative risk of injury in the following week. The ACWR has been utilised as an outcome measure to monitor an athlete’s training load over time ^[7]^. Moderate ACWRs combined with a high chronic workload overall have been found to decrease the risk of injury ^[7]^. Thus, a higher chronic workload increases the athlete’s injury threshold and therefore serves as a protective factor against injury ^[5]^.

An ACWR of between 0.8 and 1.3 has been proposed to reduce injury risk in team sport athletes such as rugby, soccer and cricket. If an athlete’s ACWR is outside of this proposed ‘sweet spot’, the risk of injury and illness is thought to increase ^[5]^. However, this relationship has not been adequately established in ultramarathon runners. Recently, the ACWR has been criticised for its mathematical flaws ^[8]^. Instead, the use of the exponentially weighted moving averages (EWMA) in the calculation of the ACWR has been proposed as it appears to be more sensitive in determining spikes in training loads ^[9]^.

Understanding the relationship between training loads, injury and illness profiles of ultramarathon runners may prove to be beneficial in terms of minimising the risk of injury and illness and maximising performance in these athletes ^[10]^. The authors therefore aim to add to current literature with regard to injury and illness profiles of ultramarathon runners, as well as the use of internal training loads, external training loads and the EWMA of the ACWR in the monitoring and prevention of injury and illness in endurance runners.

## Methods

### Ethical considerations

Ethical approval was obtained from the University of Cape Town, Faculty of Health Sciences Human Research Ethics Committee prior to the start of the study.

### Participants

This prospective, descriptive, longitudinal study design included 119 ultramarathon runners who qualified for the 2019 Two Oceans ultramarathon race. Those with relevant medical or surgical history that would prevent safe participation were excluded from this study. Those who did not complete the informed consent form and those who sustained a running-related injury in the seven day period prior to the start of the study were also excluded.

### Measurement instrumentation

#### Physical activity readiness questionnaire

The Physical Activity Readiness Questionnaire (PAR-Q+) was used to screen participants for any potential underlying medical and surgical conditions that may limit safe participation in physical activity. Participants who answered ‘no’ to all initial questions were cleared to take part in the study. Participants who answered ‘yes’ to one or more of the initial questions then had to complete the follow-up questions. If they further answered ‘yes’ to one or more of the follow-up questions, participants were excluded from the study and advised to seek medical help ^[11]^.

#### Baseline questionnaire

A self-developed questionnaire was used to establish participants’ training, injury and illness history. The questionnaire was reviewed by a panel of experts to establish content and construct validity. Once the validation process was completed, the feasibility of the questionnaire was assessed through a pilot study of four participants. Data from pilot study participants were not included for analysis, as they completed the baseline questionnaire only.

#### Training injury and illness logbook

Participants kept a weekly logbook of their training, injury and health information for the 16-week study period, which comprised 12 weeks before the race and four weeks after the race. Training information included average training distance (km.week^−1^), duration (min.week^−1^), and frequency (sessions.week^−1^). Participants also recorded average weekly rate of perceived exertion (RPE), using the modified Borg scale ^[12]^. Participants indicated if they had sustained a running-related injury each week. Participants who sustained injuries recorded if the injury was new or recurrent, the injury type, as well as the site of pain. Time-loss from running was also recorded. Participants documented their weekly health status and if they had contracted any new or recurring illnesses. Participants who reported an illness were requested to document the associated symptoms, the influence of the illness on their participation in training and any treatment that was received for the illness. The logbook was distributed via email on Sunday of each week for 16 weeks which contained a link to SurveyMonkey for completion.

### Data analysis

Injury incidence was determined according to the authors’ definition of a running-related injury which was taken from the 2015 consensus statement (a modified Delphi approach) ^[13]^. A running-related injury was therefore constituted as; ’Running-related (training or competition) musculoskeletal pain in the lower limbs that causes a restriction on or stoppage of running (distance, speed, duration, or training) for at least seven days or three consecutive scheduled training sessions, or that requires the runner to consult a physician or other health professional ‘^[13]^. A new injury was defined as any new area of pain and/or the recurrence of a previous injury with more than a week’s gap in symptoms. A recurring injury was defined as the same injury that the participant had experienced in the previous week. Injury proportion was calculated as the number of injured participants divided by the total sample size. Injury incidence was calculated as the number of injuries per 1000 hours of training.

Illness incidence was determined as ’A new or recurring illness incurred during competition or training receiving medical attention, regardless of the consequence with respect to absence from competition or training’ ^[14]^. A new illness was defined as illness-related symptoms that the participant had not experienced in the previous week, whereas a recurring illness pertained to illness-related symptoms that the participant had experienced in the previous week. Illness proportion was calculated by dividing the number of ill participants by the total sample size. Illness incidence was calculated as the number of ill participants per 1000 training days.

### Statistical analysis

Descriptive characteristics of the study population were described using mean and standard deviation. Differences in descriptive data between the groups were assessed using an independent t-test. Training parameters during the 16-week study period were described using mean and standard deviation for both the injured and uninjured groups, and the ill and healthy groups.

Weekly and cumulative absolute and relative training load parameters were described using mean and standard deviation for the two groups. The significant difference in the training parameters between the groups was measured using an independent t-test. Pearson’s correlations and odds ratios were used to establish associations between injury, illness, and absolute and relative training load variables. Statistical significance was accepted as p < 0.05.

## Results

At baseline, there were 75 males and 43 females. After the study was completed, participants were divided into an injured (n=37) and uninjured group (n=82); and an ill (n=79) and a healthy group (n=40), based on individual injury or illness reported during the 16-week study period. The average age of participants was 41± 10 years, the average stature was 174.5 ± 9.4 cm, the average body mass was 71.3 ± 12.6 kg and the average BMI was 23.4 ± 2.3 kg/m^2^.

### Injury profile

The overall injury proportion was 31% and the injury incidence was 5 per 1000 hours of training. The average time-loss from injury was 3 ± 2 missed training sessions. The most commonly injured structure was muscle (37%). The knee was the most common site of pain (19%), followed by the foot (14%), hip (12%) and ankle (12%).

### Illness profile

The overall illness proportion was 66% and the illness incidence was 16 per 1000 training days. The average time-loss due to illness was 3 ± 1 training sessions missed. The main illness-related symptoms that were reported were congestion (54%) and fatigue (20%).

### Training parameters

No significant difference was found between the injured and uninjured group in average cumulative distance and average cumulative duration per week. A significant difference was found between the two groups for average frequency (p=0.0286) and average sessional RPE per week (p=0.0030) (Table 1). On average, the uninjured group ran significantly more times per week than the injured group and at a significantly higher intensity.

A moderate, significant negative correlation was found between average external training load and injury incidence (r=−0.56; p=0.025). As the external training load decreased the injury incidence increased. No correlation was found between average internal training load and injury incidence. No correlation was found between average external training load and average internal training load and illness incidence.

No significant relationships were found for internal training load and injury incidence and internal training load and illness incidence for those who ran less than 30 km.wk^−1^. A significant relationship was found for external training load and injury incidence in weeks five to eight for those who ran less than 30 km.wk^−1^. (Table 2). Significant relationships were also found for external training load and illness incidence in weeks five to eight, nine to 12 and 13 to 16 for those who ran less than 30 km.wk^−1^. (Table 3).

A significant relationship was found for the ACWR and injury incidence in weeks one to four, five to eight and 13 to 16 when the ACWR was >1.5 (Table 4). A significant relationship was found for the ACWR and illness incidence in weeks 13 to 16 when the ACWR was <0.05 (Table 5).

## Discussion

A total of 37 participants among the 119 participants sustained a running-related injury over the 16-week study time period, indicating an incidence proportion of 31%. The overall injury incidence was five per 1000 hours of training. The authors used the 2015 consensus statement which identified running-related injuries through a modified Delphi approach ^[12]^.

A running-related injury was classified according to time-loss (three or more missed training sessions) ^[12]^. According to Clarsen and Bahr the ‘time-loss’ definition of an injury is both reliable and easy to use amongst coaches and athletes and therefore does not require the expertise of a healthcare professional ^[15]^. However, this definition also has several limitations. Many injuries may be missed as athletes often continue to train despite having an injury. Often these injuries are not serious enough to warrant stopping training but rather managed through load modification, such as reducing the length or intensity of the exercise session and/or through the use of certain over the counter medications ^[15]^.

In this study, the anatomical area most commonly injured was the knee (19%), followed by the foot, hip, ankle and hamstring. Results from this study are consistent with many other studies which have reported the lower limb to be the most commonly injured area of the body, more specifically the knee ^[16]^.

Imbalances in the lower limb may contribute to the development of running-related injuries ^[17]^. The hip serves as a dynamic stabiliser of the lower limb. Weakness of the hip stabilisers, such as the hip abductors and hip external rotators, has been found in patients with patellofemoral pain syndrome and iliotibial band friction syndrome ^[17]^. Weak hip abductors increase the amount of adduction occurring at the hip which increases the angle of pull on the knee ^[17]^. This is supported by Ramskov et al. who found greater hip abductor strength to be associated with less patellofemoral pain ^[18]^. Other biomechanical abnormalities that have been associated with an increased risk of knee pain include knee malalignment, excessive pronation and an increased Q-angle ^[18]^.

A total of 79 participants among the 119 participants sustained an illness over the 16-week study time period, indicating an incidence proportion of 66%. The overall illness incidence in this study was 16 per 1000 training days. Findings from this study vary with illness incidences found in other studies which could be due to differences in illness definitions and periods of data collection ^[14]^.

The main illness-related symptom reported in this study was congestion (54%). Schwellnus et al. found that 50% of illnesses reported by athlete’s effect the respiratory tract ^[14]^. Symptoms of an upper respiratory tract infection include a sore throat, congestion and cough ^[14]^. Following an acute bout of stress (i.e. an ultramarathon), the immune system is believed to be suppressed ^[4]^. It is during this period of decreased immunity that the risk of sustaining an upper respiratory tract infection increases ^[4]^.

A significant negative association was found between external training load and injury (r=−0.56; p=0.025). As the external training load decreased the incidence of injury increased. Gabbett found that increasing one’s overall training load improves performance ^[5]^. In individual sports, such as running, an association between higher chronic training loads and improved performance has been established ^[5]^. Gabbett and Whiteley have suggested that if an athlete is loaded beyond the specific requirements of an event or match (i.e. by increasing their overall chronic load) then their risk of injury can be minimised during these high periods of stress and in turn increase an athlete’s injury threshold ^[10]^.

An ACWR of 0.8 to 1.3 has been recommended as the ‘sweet spot’ in training load prescription, above and below which the relative risk of injury increases ^[5]^. However, this is not specific to ultramarathon running. A significant relationship was found in these authors’ study when examining the ACWR and illness incidence when the ACWR was <0.5. These findings indicate that a lower training mileage was associated with an increased risk of illness. However, this was found in weeks 13 to 16 after the ultramarathon event took place. Thus, this more likely indicates that participating in an ultramarathon increases the risk of illness post event as opposed to a low training mileage being associated with an increased illness incidence.

When the ACWR was ≥1.5 in these authors’ study, a significant relationship was found between the ACWR and relative injury risk. An ACWR of >1.5 is suggestive of a sudden ‘spike’ in an athlete’s training load ^[5]^. It is at this point that the risk of injury in the following week starts to rise. The use of the ACWR to monitor these sudden spikes in training load as well as determining the athlete’s overall chronic load on a four weekly basis has proven to be effective ^[9]^. Both rolling averages and the exponentially weighted moving average have been used in the calculation of the ACWR in literature. The EWMA method of calculation gives more weighting to recent training loads towards the end of a four week training block and lesser weighting to older values ^[9]^. It appears to be more sensitive to changes in the chronic load as well as predicting signs of fatigue. It is therefore recommended in the calculation of the ACWR ^[9]^.

The results from this study add to the current knowledge-base on the use of the ACWR in ultramarathon runners specifically. For individual athletes, an ACWR of 0.5 to 1.5, using the EWMA method of calculation, may be appropriate in terms of minimising the risk of injury and illness in the following week until such time as more research is established.

## Conclusion

In conclusion, a lower training load could potentially predispose to running-related injuries or the development of illness. Specifically, a weekly mileage of less than 30 km per week may increase the risk of sustaining an injury or illness when training for an ultramarathon event. An ACWR greater than 1.5 may increase the risk of injury in the subsequent week of training and an ACWR less than 0.5 may increase the risk of illness in the following week. Non-gradual changes to a weekly training load, whether increases or decreases, could increase the risk of incurring a running-related injury or illness. Maintaining an ACWR between 0.5 and 1.5 appears to be optimal in minimising the risk of sustaining a running-related injury or illness. These authors recommend the use of both absolute (internal and external) and relative workloads (EWMA of the ACWR) in the monitoring of an athlete’s training load with the aim of minimising injury and illness risk and maximising performance in ultramarathon runners.

## Figures and Tables

**Table 1:** Training parameters (average distance, average duration, average frequency and average session RPE) between the total, uninjured and injured groups over the 16-week study time period

	Total group (n=119)	Uninjured (n=82)	Injured (n=37)	t-value	p-value
**Distance (km.wk** **^−1^** **)**	52 ± 38	54 ± 31	44 ± 22	1.74	0.08
**Duration (min.wk** **^−1^** **)**	132 ± 129	126 ± 83	128 ± 88	0.13	0.89
**Frequency (sessions.week** **^−1^** **)**	4 ± 2	4 ± 1	3 ± 1	2.23	0.0286*
**Average session RPE (0–10)**	4 ± 2	5 ± 1	4 ± 1	3.07	0.0030**

Data are expressed as mean ± standard deviation (SD).

* indicates p<0.05 significant difference;

** indicates p<0.001 significant difference.

RPE, rate of perceived exertion.

**Table 2:** Odds ratio (95% CI) for risk factors for injury incidence and external training loads

External	Week 1 to 4 (Early training)		Week 5 to 8 (Mid training)		Week 9 to 12 (Pre-race)		Week 13 to 16 (Post-race)	

Exposure group (km.wk^−1^)	Odds ratio (95% CI)	p-value	Odds ratio (95% CI)	p-value	Odds ratio (95% CI)	p-value	Odds ratio (95% CI)	p-value
≥0 – <30	1.3 (0.6 – 2.7)	0.46	9.7 (2.0 – 46.8)	0.0047**	3.4 (0.9 – 11.6)	0.05	2.2 (0.2 – 19.8)	0.49
≥30 – <60	1.0		1.0		1.0		1.0	
≥60 – <90	0.6 (0.3 – 1.4)	0.20	4.5 (0.9 – 22.6)	0.07	0.8 (0.2 – 3.2)	0.74	1.5 (0.1 – 24.7)	0.77
≥90–<120	0.5 (0.1 – 3.8)	0.48	0.9 (0.0 – 18.5)	0.93	0.4 (0.0 – 7.3)	0.52	1.0 (0.0 – 25.7)	0.99
≥120	2.2 (0.4 – 11.1)	0.35	2.9 (0.1 – 64.3)	0.50	3.3 (0.3 – 32.5)	0.31	3.8 (0.1 – 103.5)	0.43

* indicates p<0.05 significant difference;

** indicates p<0.001 significant difference.

Reference group **is ≥**30–<60 km.wk^−1^.

**Table 3:** Odds ratio (95% CI) for risk factors for illness incidence and external training loads

External	Week 1 to 4 (Early training)		Week 5 to 8 (Mid training)		Week 9 to 12 (Pre-race)		Week 13 to 16 (Post-race)	

Exposure group (km.wk^−1^)	Odds ratio (95% CI)	p-value	Odds ratio (95% CI)	p-value	Odds ratio (95% CI)	p-value	Odds ratio (95% CI)	p-value
≥0–<30	1.6 (0.8 – 2.9)	0.16	2.3 (1.2 – 4.6)	0.02*	2.9 (1.3 – 6.2)	0.01**	5.4 (2.3 – 12.3)	0.0001**
≥30–<60	1.0		1.0		1.0		1.0	
≥60–<90	0.5 (0.3 – 1.2)	0.11	1.0 (0.5 – 2.1)	0.94	0.8 (0.4 – 1.9)	0.66	1.1 (0.3 – 3.5)	0.93
≥90–<120	0.7 (0.2 – 3.4)	0.69	0.6 (0.2–2.1)	0.43	0.8 (0.2 – 3.0)	0.75	1.6 (0.4 – 6.5)	0.53
≥120	0.7 (0.1 – 5.7)	0.73	0.7 (0.1 – 5.8)	0.74	0.4 (0.0 – 6.9)	0.52	1.77 (0.2 – 16.2)	0.61

* indicates p<0.05 significant difference;

** indicates p<0.001 significant difference.

Reference group **is ≥**30–<60 km.wk^−1^.

**Table 4:** Odds ratio (95% CI) for risk factors for injury incidence according to acute:chronic workload ratios (ACWRs)

External	Week 1 to 4 (Early training)		Week 5 to 8 (Mid training)		Week 9 to 12 (Pre-race)		Week 13 to 16 (Post-race)	

ACWR ratios	Odds ratio (95% CI)	p-value	Odds ratio (95% CI)	p-value	Odds ratio (95% CI)	p-value	Odds ratio (95% CI)	p-value
≥0.0 – <0.50	3.1 (0.9 – 9.8)	0.06	1.0 (0.3 – 3.9)	0.99	0.4 (0.1 – 1.8)	0.22	0.7 (0.2 – 3.2)	0.64
≥0.5 – <1.0	0.9 (0.2 – 3.5)	0.88	1.4 (0.5 – 4.3)	0.55	0.4 (0.1 – 1.5)	0.18	0.9 (0.2 – 4.2)	0.90
≥1.0 – <1.5	1.0		1.0		1.0		1.0	
≥1.5 – <2.0	15.2 (3.9 – 59.8)	0.0001**	0.9 (0.1 – 17.2)	0.96	1.9 (0.2 – 16.2)	0.57	5.7 (1.2 – 27.5)	0.03*
≥2.0	10.7 (0.4 – 282.9)	0.16	31.0 (1.8 – 547.9)	0.02*	6.5 (0.3 – 171.6)	0.26	34.1 (0.6 – 1920.1)	0.09

* indicates p<0.05 significant difference;

** indicates p<0.001 significant difference.

Reference ratio is ≥1.0 – < 1.5.

**Table 5:** Odds ratio (95% CI) for risk factors for illness incidence according to acute:chronic workload ratios (ACWRs)

External	Week 1 to 4 (Early training)		Week 5 to 8 (Mid training)		Week 9 to 12 (Pre-race)		Week 13 to 16 (Post-race)	

ACWR ratios	Odds ratio (95% CI)	p-value	Odds ratio (95% CI)	p-value	Odds ratio (95% CI)	p-value	Odds ratio (95% CI)	p-value
≥0.0 – <0.50	1.5 (0.7 – 3.4)	0.31	1.8 (0.8 – 4.1)	0.17	1.3 (0.5 – 3.7)	0.64	2.1 (1.0 – 4.3)	0.038*
≥0.5 – <1.0	1.1 (0.6 – 2.0)	0.89	1.0 (0.5 – 1.9)	0.98	1.6 (0.9 – 3.1)	0.13	1.6 (0.8 – 3.2)	0.17
≥1.0 – <1.5	1.0		1.0		1.0		1.0	
≥1.5 – <2.0	2.1 (0.6 – 8.3)	0.27	1.8 (0.5 – 6.8)	0.39	1.8 (0.4 – 8.8)	0.47	2.4 (0.8 – 7.3)	0.13
≥2.0	6.3 (0.1 – 324.7)	0.36	1.3 (0.1 – 27.6)	0.87	8.8 (0.2 – 455.8)	0.28	7.5 (0.1 – 388.8)	0.32

* indicates p<0.05 significant difference;

** indicates p<0.001 significant difference.

Reference ratio is ≥1.0 – < 1.5.
